# Sub-Internal Limiting Membrane Hemorrhage: Molecular Microenvironment and Review of Treatment Modalities

**DOI:** 10.3390/ijms27031336

**Published:** 2026-01-29

**Authors:** Krzysztof Eder, Paulina Langosz, Marta Danikiewicz-Zagała, Rafał Leszczyński, Dorota Wyględowska-Promieńska

**Affiliations:** 1 Department of Ophthalmology, Prof. Kornel Gibiński University Clinical Center, Medical University of Silesia, ul. Ceglana 35, 40-514 Katowice, Poland; plangosz@uck.katowice.pl (P.L.); mdanikiewicz@uck.katowice.pl (M.D.-Z.); rafles3@wp.pl (R.L.); 2 Department of Ophthalmology, Faculty of Medical Sciences in Katowice, Medical University of Silesia, ul. Ceglana 35, 40-514 Katowice, Poland

**Keywords:** sub-internal limiting membrane hemorrhage, oxidative retinal injury, vitrectomy, laser membranotomy

## Abstract

Sub-internal limiting membrane (sub-ILM) hemorrhage is a distinct preretinal bleeding entity in which blood accumulates between the ILM and the retinal nerve fiber layer (RNFL), forming a sharply confined compartment. The ILM’s low permeability and lack of immune cell access create a stagnant microenvironment in which erythrocyte lysis leads to the accumulation of hemoglobin, heme, and iron, promoting the generation of reactive oxygen species. This oxidative burden poses a direct risk to retinal ganglion cells and Müller cell endfeet. Spectral-domain optical coherence tomography (SD-OCT) enables precise identification of sub-ILM blood through its characteristic dome-shaped elevation and hyperreflective contents, distinguishing it from subhyaloid and vitreous hemorrhage. Management options include observation, neodymium-doped yttrium–aluminum–garnet (Nd: YAG) laser membranotomy, pneumatic displacement, and pars plana vitrectomy (PPV). While small, extrafoveal hemorrhages may resolve spontaneously, prolonged blood entrapment is associated with increased retinal toxicity, tractional changes, and proliferative vitreoretinopathy (PVR). Early intervention generally results in faster clearance and improved visual outcomes, particularly for dense or foveal bleeding. Major gaps remain regarding cellular stress responses, biomarkers that predict irreversible damage, and the optimal timing of intervention. Standardized imaging criteria and evidence-based management algorithms are needed to guide individualized treatment.

## 1. Introduction

### 1.1. Definition and Clinical Importance of Sub-ILM Hemorrhage

Sub-internal limiting membrane (sub-ILM) hemorrhage is defined as the presence of extravasculated blood between the retina’s outermost layer of internal limiting membrane and further interiorly lying retinal nerve fiber layer. This potential space, when filled with blood, forms a dome-shaped, closed compartment, sharply demarcated by the elevation of the ILM [1]. Specific optical opacity, often involving the macula, constitutes the classical fundus finding on physical examination. Clinically, sub-ILM hemorrhage, recognized by some authors as a subtype of vitreous hemorrhage rather than a retinal bleeding, presents with acute or subacute unilateral painless vision loss, central scotoma, or acute-onset metamorphopsias, depending on the scope and location of the pathology [2].

Relatively uncommon as compared to classical intravitreal vitreous hemorrhage or subhyaloidal hemorrhage, sub-ILM hemorrhage is also usually recognized in a specific co-morbidity context of Valsalva retinopathy, Terson syndrome, ocular trauma [3], or rupture of fragile neovascular vessels [4,5]. Estimated frequency of the abovementioned etiologies, along with their specific clinical and pathophysiological profiles are presented in Table 1. Due to the structural rigidity of the ILM, the blood accumulated underneath does not display a tendency for migration into the vitreous gel, providing a fine confinement of the extravasculated blood in the direct vicinity of the retinal nerve fibers [6]. This, in turn, raises a valid question about the biochemical microenvironment created by the trapped hemorrhage, along with treatment’s potential for preventing neuroretinal damage [7].

### 1.2. Distinction Between Sub-ILM, Subhyaloid, and Intravitreal Hemorrhages

Sub-ILM hemorrhage must be differentiated from subhyaloidal and classical vitreous hemorrhages, as each occurs in a distinct anatomical compartment with diverse optical, physiological, and biochemical consequences. All of the above can, however, co-occur [8].

Subhyaloid hemorrhage, as opposed to sub-ILM bleeding, locates itself in between the posterior hyaloid and the ILM membrane, typically forming a boat-shaped, even fluid level. Posterior hyaloid has been proven to be more permissive than the ILM, thus allowing, in some cases, diffusion and/or spontaneous rupture of the membrane, followed by hemorrhage distribution into the vitreous gel. The latter constitutes a classical vitreous hemorrhage (VH) within which erythrocytes can diffuse, sediment, and undergo a more gradual dilution, marking a significantly different microenvironment than the one observed in sub-ILM hemorrhage, i.e., in terms of oxygen tension, enzymatic clearance capacity, and exposure to immune cells such as macrophages and hyalocytes [9].

In contrast, sub-internal limiting membrane bleeding occurs within a sealed, avascular compartment, anteriorly bounded by an almost non-permissive basement-membrane-like structure rich in collagen IV and laminins; it is a compartment lacking in macrophage access, characterized by minimal fluid turnover [10], which results in an in situ lysis of the extravasculated erythrocytes and accumulation of their products in the direct vicinity of the inner retina. Understanding these distinctions is essential for interpreting clinical course and guiding intervention.

**Figure 1:**
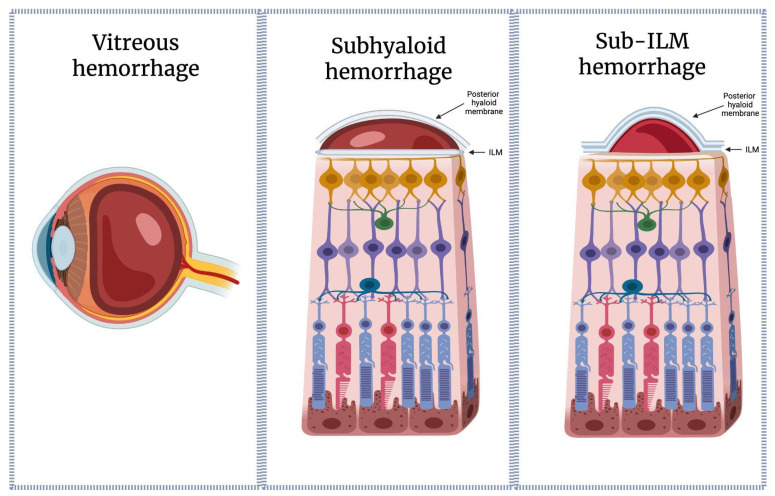
Visual representation of intravitreal, subhyaloid, and sub-ILM hemorrhages, respectively. Curated using the BioRender software. Created in BioRender. Eder, K. (2026) https://BioRender.com/h2cdk2t (accessed on 15 January 2026).

Clinically and on optical coherence tomography, subhyaloid hemorrhage characteristically demonstrates a boat-shaped configuration with a horizontal fluid level reflecting gravitational layering beneath the posterior hyaloid. Conversely, sub-ILM hemorrhage is defined by a smooth, dome-shaped elevation of the internal limiting membrane, reflecting blood entrapment within a sealed, non-dependent compartment, as presented in Figure 1.

### 1.3. Rationale for Focusing on Molecular and Microenvironmental Aspects

While the clinical presentation and treatment of sub-ILM hemorrhages are relatively well described, the molecular microenvironment created by blood trapped under the ILM has received far less attention. The ILM’s limited permeability, its intimate contact with Müller cell endfeet, and the confined nature of the sub-ILM space suggest that erythrocyte breakdown may expose the inner retina to elevated concentrations of hemoglobin, heme, and iron, as well as reactive oxidative species generated in a low-diffusion environment [11]. These molecular events may have implications for inner retinal stress, including oxidative injury to ganglion cells, altered Müller cell homeostasis, or disruption of the nerve fiber layer [12]. Many models were created as a trial of mapping the oxygen and oxygen-related stress distribution across the globe [13]. Furthermore, therapeutic decisions, especially on whether to observe or perform Nd: YAG membranotomy, attempt pneumatic displacement, or perform vitrectomy, may benefit from biochemical understanding of how long and to what extent the retina can tolerate sub-ILM blood presence.

### 1.4. Scope and Structure of the Review

In this review, we firstly outline the anatomical and molecular properties of the internal limiting membrane (ILM) that underlie the formation of a confined sub-ILM compartment. We then proceed to discuss the biochemical and cellular consequences of blood entrapment beneath the ILM, with particular emphasis placed on hemoglobin breakdown, iron-mediated oxidative stress, and inner retinal damage. Subsequently, we summarize characteristic retinal imaging features and biomarkers that reflect the temporal evolution of sub-ILM hemorrhage. Finally, we review currently available treatment modalities, linking their clinical applications to the underlying molecular microenvironment, and highlighting existing knowledge gaps as well as future research directions aimed at optimizing treatment timing and outcomes.

## 2. Anatomy and Molecular Structure of the Internal Limiting Membrane

### 2.1. Structural Components (Collagen IV, Laminins, Nidogens, Proteoglycans)

The ILM serves as the innermost surface of the retina and can be recognized as a type of specialized basal membrane. Synthesized primarily by Müller glial cells, it consists predominantly of type IV collagen, laminins, nidogens, and heparan sulfate proteoglycans, most notably perlecan. As laminins regulate adhesions between Müller cell endfeet and the extracellular matrix (ECM), type IV collagen provides tensile integrity, and nidogen-1 and nidogen-2 act as bridging molecules, linking collagen IV networks with laminins to stabilize the ILM’s three-dimensional lattice.

These molecular constituents form a highly ordered sheet, varying in thickness from ~0.2 μm in the fovea to 3 μm near the optic nerve head. This graded architecture reflects regional differences in Müller cell density, vitreoretinal adhesion, and mechanical stress distribution across the posterior pole. Importantly, the ILM exhibits low permeability to proteins and cells, functioning as a diffusion barrier that separates the vitreous from neural retinal layers.

### 2.2. Barrier Properties and Permeability Characteristics

The ILM’s molecular composition gives rise to distinct biophysical properties, including (i) selective permeability, (ii) high tensile strength, and (iii) resistance to enzymatic degradation. Studies using transmission electron microscopy and tracer experiments demonstrate that the ILM restricts the passage of large proteins (generally >70 kDa), immune cells, and most plasma components under physiological conditions [1]. The tightly cross-linked network of collagen IV, laminins, and heparan sulfate proteoglycans markedly reduces hydraulic permeability, limiting passive diffusion of fluids and solutes across the membrane [14]. In addition, the ILM lacks mechanisms for active transport or transcytosis, precluding the regulated delivery of plasma proteins into the sub-ILM space. This has direct biochemical consequences: large hemoglobin-scavenging proteins such as haptoglobin (~85–100 kDa) are unable to access sub-ILM hemorrhage, and clearance of hemoglobin–haptoglobin complexes further depends on CD163-expressing macrophages, which—although present in the cortical vitreous—cannot traverse the ILM. Consequently, the sub-ILM compartment is devoid of effective hemoglobin-scavenging and innate immune clearance mechanisms. These barrier characteristics explain both the physical confinement of hemorrhage beneath the ILM, resulting in a typical sharply demarcated dome on OCT imaging [15], and the persistence of a locally toxic biochemical microenvironment adjacent to the inner retina.

### 2.3. ILM–Müller Cell Interface

Müller glia produce the ILM via secretion of collagen IV, laminins, and proteoglycans at their endfeet, which constitute the posterior barrier of the internal limiting membrane. This interface is essential for maintaining retinal biomechanics and homeostasis: it regulates inner retinal hydration, potassium buffering, and extracellular space geometry [16]. The ILM also anchors Müller endfeet via laminin–integrin interactions (particularly α6β1 and α3β1 integrins as well as α-dystroglycan), which are important for structural stability of the nerve fiber layer and for retinal development [1]. Müller cells maintain ionic, neurotransmitter, and water homeostasis in the inner retina by actively regulating (i) potassium buffering via Kir channels, (ii) glutamate uptake via excitatory amino acid transporters (EAATs), (iii) water movement through aquaporin-4, and (iv) extracellular pH through carbonic anhydrase activity. The ILM modulates the diffusion gradients supporting these functions. Its low permeability creates a semi-isolated microenvironment between the vitreous and the nerve fiber layer, ensuring stable ionic conditions and acid–base balance for ganglion cell axons.

## 3. Molecular Microenvironment of Blood Entrapment Under the ILM

There are several iron-dependent enzymes essential for proper retinal function, inter alia: (1) guanylate cyclase, which is required for cyclic guanosine monophosphate (cGMP) synthesis in phototransduction cascade; (2) fatty acid desaturase, which produces lipids for the constantly shedding photoreceptor disc membranes; and (3) isomerohydrolase, which catalyzes the conversion of all-trans-retinyl ester to 11-cis-retinol in the visual cycle in retinal pigment epithelium (RPE) [17,18,19]. A stable supply of iron—necessary for the proper function of each of these enzymes—is maintained through transferrin-mediated endocytosis using transferrin receptors, which are located inter alia in the RPE, ganglion cell layer, inner nuclear layer, outer plexiform layer, inner segment of photoreceptors, and choroid [20]. The RPE’s transferrin receptors are located on both basolateral and apical surfaces, suggesting that there is a bidirectional iron movement across the RPE cells [21]. Inside the cell, iron is separated from transferrin due to low pH in the endosomes and released to the cytoplasm for usage, storage, or export. Intracellular iron is mostly stored by ferritin, while the metabolically active iron is mostly used in mitochondria. The excess iron can be exported in its ferrous state by ferroportin, but it requires ceruloplasmin or hephaestin for oxidation to be accepted by circulating transferrin again [22].

**Figure 2:**
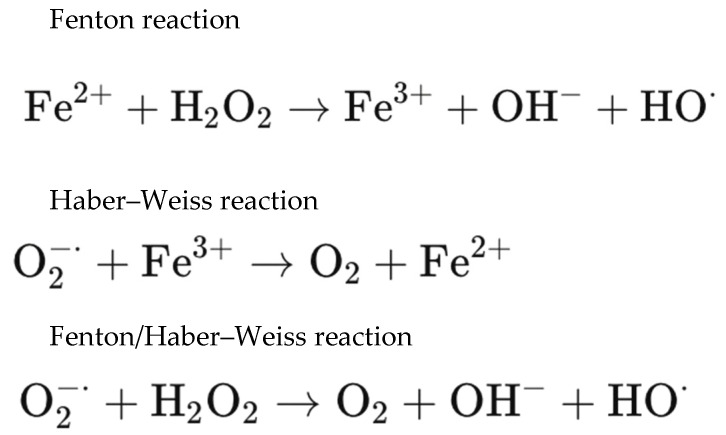
Radical-forming redox reactions implicated in hemoglobin-mediated oxidative damage in sub-ILM hemorrhage.

The versatility and importance of iron ions in retinal metabolic processes derive from their ability to easily cycle between Fe^2+^ and Fe^3+^ forms while donating or accepting electrons, respectively. The same redox activity, however, also drives oxidative injury via the Fenton/Haber–Weiss reaction, as presented in Figure 2. In the presence of iron ions, hydrogen peroxide is catalyzed to the hydroxyl free radical, which amplifies oxidative stress of retina, causing inter alia DNA damage, lipid peroxidation, and the breakdown of essential biomolecules [13,17,22,23]:

The retina is especially susceptible to oxidative stress due to high oxygen tension caused by high perfusion combined with continuous light exposure, photo-oxidative load, and the presence of easily oxidized lipids in photoreceptor outer segments. The peroxidation can proceed due to reactive oxygen species as well as chain propagation induced by Fe^2+^ without directly involving the hydroxyl radicals or other active oxygen species [23,24]. Studies further suggest that the combination of high concentration of Fe^2+^ and outer disc segments of photoreceptors is particularly injurious to RPE, exceeding the peroxidation toxicity of phagocytosed photoreceptor outer segment discs [25].

Iron, especially in the Fe^2+^ state, can also promote protein modification by bisretinoid photofragments, leading to further cellular damage [26]. Additionally, in mammalian cell cultures, high concentrations of Fe^2+^ significantly affected the permeability of cells, and the effect increased throughout the exposure, facilitating Fe^2+^ diffusion and expanding the intracellular labile iron pool [27]. Elevated intracellular iron, in turn, impairs RPE phagocytosis and lysosomal function, further undermining photoreceptor support [25,28]. Free heme that is released during erythrocyte in situ lysis directly activates pattern-recognition receptors, particularly the toll-like receptor 4 (TLR4) on endothelial cells and macrophages. Heme-driven redox cycling and iron release cause oxidative stress that leads to mitochondrial dysfunction and activation of mitogen-activated protein kinases (MAPK) and nuclear factor kappa-light-chain-enhancer of activated B cells (NF-κB) signaling pathways. Simultaneously, ROS initiate the assembly of the NLR family pyrin domain containing 3 (NLRP3) inflammasome. This results in caspase-1 activation and IL-1β and IL-18 maturation. Synchronously, these pathways cause robust production of inflammatory cytokines such as TNF-α, IL-6, and IL-1β, as well as chemokines such as CXCL8 and monocyte chemoattractant protein-1 (MCP-1/CCL2). In parallel, an anti-inflammatory counter reaction is induced by the production of IL-10 and TGF-β.

**Figure 3:**
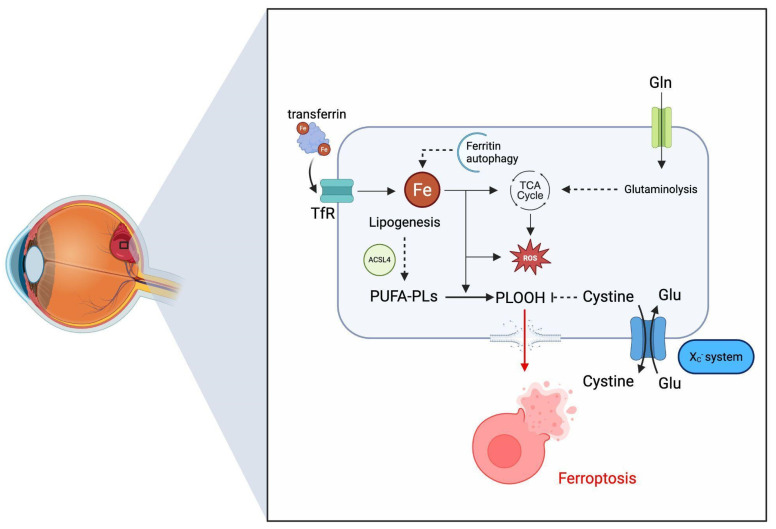
Simplified visual representation of a sub-ILM ferroptosis pathway. Curated using the BioRender software. Created in BioRender. Eder, K. (2026) https://BioRender.com/55g3mqt (accessed on 15 January 2026).

Moreover, the accumulation of processes described above can lead to ferroptosis. Ferroptosis is an iron-dependent type of non-apoptotic cell death characterized by the accumulation of lipid peroxides, initiated by an increase in the labile intracellular iron pool. Biochemical and cellular mechanisms associated with the process are visualized in Figure 3. The process may arise, among others, from an enhanced iron reuptake by means of the Transferrin—Transferrin Receptor (TfR) pathway. Elevated levels of ferrous iron (Fe^2+^) initiate the generation of reactive oxygen species (ROS) through the Fenton reaction, thereby creating a pro-oxidative intracellular environment. During the process, polyunsaturated fatty acids (PUFAs) are incorporated into membrane phospholipids through the activity of acyl-CoA synthetase long-chain family member 4 (ACSL4), significantly increasing susceptibility of the membranes to oxidative damage, leading to enzymatic and non-enzymatic oxidation. The process leads to the accumulation of phospholipid hydroxyperoxides (PLOOHs), resulting in antioxidant defense failure. Antioxidant activity is compromised by either depletion of intracellular glutathione, direct activation of glutathione–glutathione peroxidase 4 (GPX4), or through inhibition of cystine/glutamate antiporter system X_C_^−^. Antioxidant activity may also be compromised by failure of the auxiliary defense mechanisms, such as the FSP1–coenzyme Q10–NAD(P)H system. Mitochondrial metabolism also contributes to ferroptosis. The active tricarboxylic acid (TCA) cycle sustains glutaminolysis-driven anaplerosis and ROS production, therefore increasing lipid peroxidation, under conditions of compromised antioxidant defense. Those mechanisms induce uncontrollable lipid peroxidation, leading to membrane rigidity and rupture with imminent ferroptotic cell death and the release of intracellular contents, which, in turn, provokes an inflammatory response [29].

As such, iron overload caused by sub-ILM hemorrhage can cause significant damage. Beyond the iron-mediated oxidative injury to retinal cells, the mechanical effects of a dense clot—elevated local pressure, disruption of nutrient exchange, and shear injury to inner retinal layers—can also contribute to tissue damage [17]. Sub-ILM hemorrhage is also associated with increased retinal toxicity, higher risk of premature epiretinal membrane or macular hole formation, and proliferative vitreoretinopathy [30]. In cases managed with the PPV, the peeled ILM was extracted for histological examination [31]. It revealed macrophages with phagocytosed hemosiderin, retinal pigment epithelial and glial cells, and inflammatory monocytes and fibroblasts on the retinal side of the ILM. This suggests an active, ongoing process to repair the damaged tissue on the retinal side of the ILM. At the same time, no such cells were observed on the vitreous side [1,6,18,21].

## 4. Diagnostic Features and Imaging Biomarkers

### 4.1. Sub-ILM Hemorrhage SD-OCT Imaging Characteristics

**Figure 4:**
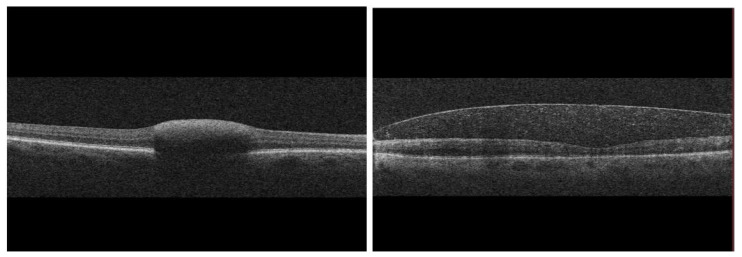
Comparison of sub-ILM hemorrhage (**left**) and subhyaloid hemorrhage (**right**) on SD-OCT.

SD-OCT, along with a wide range of other applications, has, over the recent years, become the gold standard for localization of pre- and intraretinal hemorrhage surfaces. The Sub-ILM hemorrhage typically appears as a dome-shaped elevation of the ILM, forming a smooth, hyperreflective boundary overlying a pocket of highly reflective or heterogeneous material corresponding to the blood, trapped in the sub-ILM space [32,33]. The explicit differences in the OCT appearance of sub-ILM vs. subhyaloid hemorrhage are shown below in Figure 4.

Some of the main OCT findings typically include (1) a smooth ILM contour overlying the hemorrhage itself, often with a thin, continuous hyperreflective line representing the ILM; (2) dense homogeneous or polygranular reflectivity beneath the ILM, representing erythrocytes and hemoglobin degradation products; (3) sharply demarcated borders, consistent with the impermeable nature of the ILM; (4) absence of posterior hyaloid separation immediately over the dome (unless concomitant posterior vitreous detachment is present) [34,35,36,37]. So far, no clinically significant OCT biomarker has been established as a quantitative trigger point for surgical intervention. In contrast, subhyaloid hemorrhage shows a more triangular or boat-shaped configuration, bounded superiorly by the posterior hyaloid, which appears as a more undulating and less tightly adherent membrane [38]. Several OCT studies demonstrate that the structural confinement imposed by the ILM leads to an optically dense pocket that often persists until the ILM is breached, either spontaneously or through intervention [4,14,39]. The imaging biomarkers of specific phases of sub-ILM hemorrhage evolution, along with their biochemical correlates are pooled in Table 2.

### 4.2. Autofluorescence, Fluorescein Angiography and Near-Infrared Signatures

Autofluorescence (AF) imaging provides complementary information about hemoglobin breakdown. As erythrocytes degrade, products such as methemoglobin, hemichromes, and heme aggregates can alter intrinsic fluorescence patterns.

**Figure 5:**
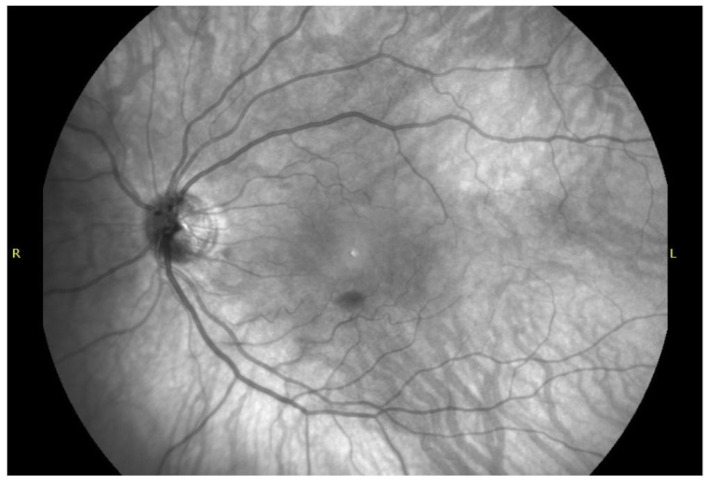
Near-infrared image of sub-ILM hemorrhage. Patient’s right side (R) on the left.

**Figure 6:**
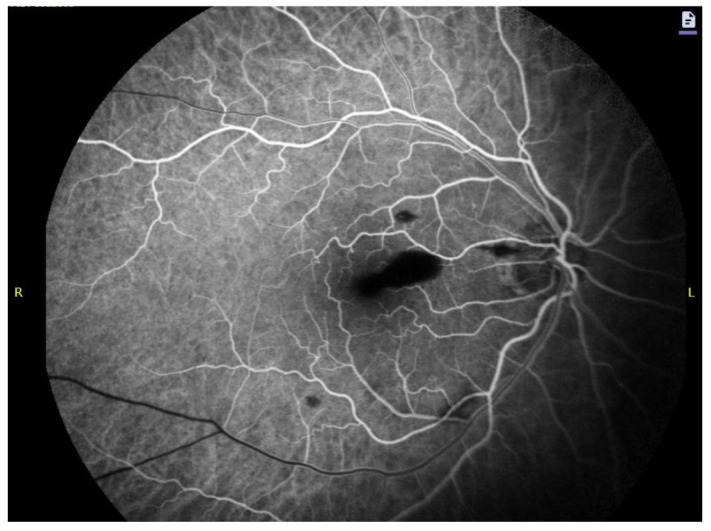
Fluorescein angiography in an eye with sub-ILM hemorrhage, early phase. Noticeable, well-defined blockage of fluorescence in the areas of a hemorrhage. Patient’s right side (R) on the left.

Characteristic findings include (i) hypoautofluorescence corresponding to dense, fresh blood; (ii) patchy or speckled hyperautofluorescence appearing with progressive hemoglobin oxidation, due to fluorescence from hemichromes and iron-bound porphyrin structures; (iii) a sharply outlined AF border, reflecting the ILM barrier. Near-infrared reflectance (NIR) imaging can further highlight the smooth curvature of the ILM and enhance visualization of the hemorrhage’s geometric boundaries, as presented in Figure 5. In fluorescein angiography (FA), sub-ILM hemorrhage presents as a well-defined area of hypofluorescence caused by the blockage of the underlying retinal and choroidal fluorescence by the presence of blood. This is distinctively visualized in Figure 6. Hypofluorescence is evident from the early phases of FA and remains unchanged in late phases with no signs of fluorescein leak or late staining. Borders are usually sharp, well-defined, and round or dome-shaped. Although AF, FA, and NIR cannot independently distinguish sub-ILM from subhyaloid hemorrhage, the consistency of the dome-shaped contour across modalities increases diagnostic confidence, the lack of which may lead to perilous conclusions at times [40].

### 4.3. Imaging Biomarkers of Hemoglobin Breakdown and Chronology

Several imaging features correlate with the biochemical state of sub-ILM blood: (i) increasing granularity or heterogeneity on OCT suggests ongoing erythrocyte lysis and hemoglobin oxidation, (ii) alterations in AF patterns may correlate with the shift from oxyhemoglobin to methemoglobin, and (iii) transition from homogeneous to layered reflectivity may signal partial resorption or clot contraction. Such correlations have been documented in preretinal and subretinal hemorrhages and are likely applicable to sub-ILM bleeds, although direct biochemical equivalence has not been established. The unique constraint of the ILM permits chronologic staging of sub-ILM hemorrhage, where imaging signatures closely reflect the microenvironmental evolution described in Section 3.

## 5. Review of Available Treatment Modalities

### 5.1. Molecular Rationale for Early vs. Delayed Intervention

There is controversy concerning the best management of isolated sub-ILM hemorrhage, ranging from conservative treatment to observation, to surgical techniques. ILM creates biochemical confinement where erythrocytes undergo in situ lysis, releasing hemoglobin degradation products, reactive oxygen species (ROS), and hydroxy radicals [1]. Toxic components remain localized adjacent to the nerve fiber layer (NFL), RGCs, and Müller cell axons. The literature shows that longer waiting time can lead to the formation of fibrous tractional membranes and proliferative vitreoretinopathy (PVR) [4]. Extended compression harm can also cause additional damage [6]. This implies that the duration of this entrapment may potentially be a negative factor in the treatment outcome concerning visual acuity and retinal function. Therefore, treatments that evacuate the sub-ILM compartment rapidly and shorten retinal exposure to potentially toxic products may be beneficial in preserving retinal function, contributing to better visual outcomes.

Observation sometimes allows for spontaneous resolution of the sub-ILM hemorrhage, but is usually slow, mainly concerning small (no larger than one disc diameter), extrafoveal hemorrhages in younger patients [41]. In the pooled literature, clearance frequently requires weeks to months, particularly for dense macular hemorrhages. In review, analysis observation produced the smallest mean visual improvement and the longest clearance time, consistent with the hypothesis that untreated sub-ILM hemorrhage may expose the inner retina to a more prolonged biochemical injury by hemoglobin and its catabolites. Observation is, therefore, appropriate primarily for small, non-macular or parafoveal and rapidly resolving hemorrhages, with little to no visual impairment, where the risk of toxicity is lower. Additionally, observation may be considered the best possible alternative in patients with contraindications to surgery, such as acute leukemia [42].

### 5.2. Immediate Intervention Overview: ILM Puncture, Pneumatic Displacement, or Vitrectomy

ILM puncture through pulses of Nd: YAG (neodymium: yttrium–aluminum–garnet) laser creates a small opening in the ILM, resulting in the immediate decompression of the sub-ILM compartment and allowing the blood to drain into the vitreous cavity [43,44]. Mechanistic consequences include rapid evacuation of potentially toxic components into the vitreous cavity and reduction of the inner retina’s exposure to oxidative intermediates. Indications for membranotomy include fresh (usually <1–2 weeks) hemorrhage, foveal location, and impairment of visual acuity. In choosing membranotomy, the duration of the hemorrhage should not be taken into account as much as the state of the blood [45]. Clotting of the blood and dense hemorrhage are contraindications for Nd: YAG membranotomy. The literature also presents case reports of patients treated with argon-laser-assisted puncture, which may present as an alternative to Nd: YAG laser treatment [46]. The method allows for rapid visual restoration and poses a safer alternative to classic surgical methods by being minimally invasive in character [47].

One of the alternative surgical treatments for sub-ILM hemorrhage is pneumatic displacement using intravitreal gas with or without tissue plasminogen activator (tPA) [48]. Tissue plasminogen activator catalyzes the conversion of plasminogen to plasmin, thus contributing to the dissolution of blood clots [49]. If the bleeding is recent and the blood is not clotted, there are no indications for the use of tPA, and the hemorrhage may be dislodged by pneumopexy alone. Similar to membranotomy, pneumatic displacement offers radio dissolution of sub-ILM hemorrhage and shortens the time of retinal exposure to toxic agents, potentially aiding in the improvement of long-term visual outcomes.

Vitrectomy with ILM peeling is a widely used surgical form of treatment of even dire cases of vitreoretinal junction pathologies [50], including the sub-ILM hemorrhages [51,52,53]. Treatment with vitrectomy allows for immediate symptom regression and prevention of blood metabolite-related conditions [54]. Studies also suggest that the technique or even restrain from carrying out ILM peeling may be a factor in the outcomes [55]. It has been shown that the foveal-sparing (button-hole) ILM-peeling technique leads to better outcomes concerning visual acuity than conventional ILM-peeling [56]. ILM lies immediately adjacent to the Müller glial cells’ endfeet and essentially serves as their basement membrane. Müller cells’ endfeet are anchored to the ILM via integrin receptors and dystroglycan complexes, which bind to ILM extracellular matrix components. ILM peeling inevitably disrupts Müller cells’ endfeet. Taking into consideration this direct relationship, both structural and molecular, the foveal-sparing technique appears to be biologically advantageous in cases of sub-ILM hemorrhage, by reducing mechanical trauma, helping to maintain foveal architecture, and reducing the risk of postoperative retinal thinning, although the clinical evidence is still lacking. Although the method is invasive and poses a threat of ocular complications, vitrectomy with ILM-peeling is very effective and leaves much less blood residue in the sub-ILM space than Nd: YAG membranotomy or pneumopexy, thus possibly contributing to limiting retinal toxicity and better long-term retinal function [57]. Mechanistically, it is the most complete resolution to biochemical confinement. Due to its invasive character, this method is mainly proposed for dense macular hemorrhages. Possible complications include retinal detachment, iatrogenic retinal breaks, macular hole, or cataract [58]. Summary of the available treatment modalities with their correlating risk-advantage profiles and expected outcomes are exhibited in Table 3.

### 5.3. Potential for Preventing Inner-Retinal Toxicity

Early surgical intervention in sub-ILM hemorrhage contributes to the immediate resolution of the toxic effect of hemoglobin degradation products, ROS, and hydroxy radicals on the inner retina, as well as the resolution of mechanical retinal compression. The literature shows that delayed surgical intervention may lead to cellular proliferation on the retinal surface of the ILM and, in consequence, to proliferative vitreoretinopathy-like pathology with permanent retinal changes [59]. A conservative approach should be considered in small, extrafoveal hemorrhages with little to no VA changes, mostly in young patients. If the hemorrhage is chronic or dense, or spontaneous resolution is insufficient, early surgical intervention is beneficial.

## 6. Future Directions

### 6.1. Gaps in Understanding Sub-ILM Biochemical Dynamics

Emerging understanding of sub-ILM hemorrhage highlights important molecular, biochemical, and clinical gaps that warrant systematic investigation. In particular, the cellular responses of Muller glia, microglia, and RGCs, as well as their interplay-cellular cross-talk, remain poorly characterized. Detailed elucidation is needed regarding how these cells orchestrate inflammatory signaling and metabolic stress pathways in response to oxidative stress caused by hemoglobin degradation products, iron- and heme-mediated oxidative injury during a hemorrhage, and to extended compression causing ischemic changes in the inner retina. Furthermore, specific mechanisms of hemorrhage-induced sub-ILM and extraretinal fibrosis, including pathways involved in extracellular matrix remodeling, often leading to PVR [4] and tractional retinal complications, remain insufficiently defined. Studies should aim to identify biochemical, molecular, or imaging biomarkers that predict irreversible retinal injury or fibrotic transformation. Future work should include experimental models of blood confinement under ILM-mimicking materials to simulate biomechanical and biochemical conditions observed clinically.

### 6.2. Need for Molecular Biomarkers and Imaging Correlates

At present, no imaging-based classification exists for sub-ILM hemorrhages. Studies indicate that photoreceptor disruption due to mechanical compression effect may be a factor in the prognosis of sub-ILM hemorrhage. It has been suggested that photoreceptor layer integrity assessed by optical coherence tomography (OCT) may serve as a potential predictor of the visual outcome [1]. These observations point to a need for further investigation and standardization of OCT-based classification of retinal hemorrhages and further correlation of imaging criteria with the best possible treatment options. Further research should focus on consensus in imaging criteria and developing automated OCT identification of hemorrhage planes (e.g., sub-ILM vs. sub-hyaloid). Additionally, it would be beneficial to develop tools for automated quantitative assessment of hemorrhage density, volume, and optical properties in OCT, which could help determine the prognosis and point to a preferable, individualized treatment modality. To the best of the authors’ knowledge, no clinically significant OCT biomarker has been established as a quantitative trigger point for surgical intervention. Considering that increasing signal granularity on OCT images is linked to erythrocyte lysis, perhaps the occurrence of such within the sub-ILM hemorrhage should serve as an imaging biomarker and a trigger for invasive treatment, to prevent the main part of RGC damage. Investigation of a possible correlation between the sub-ILM hemorrhage OCT signal intensity, as well as homogeneity and long-term visual outcomes, along with the rate of secondary complications, could yield a further contribution to the understanding of the field.

### 6.3. Research Priorities for Optimizing Treatment Timing

As previously noted, there is controversy concerning the best management of sub-ILM hemorrhage, in the range from conservative treatment to observation, to surgical techniques. Even when the need for active intervention is clinically apparent, the appropriate timing for the surgical treatment remains undefined. Currently, there are no specific evidence-based guidelines regarding the time of surgical intervention or the preferred modality. The existing literature indicates that delayed surgical intervention may lead to proliferative vitreoretinopathy-like pathologies with permanent retinal changes [59,60], but the exact preferred window during which intervention would be most beneficial has not yet been established. This highlights the need for comparative effectiveness studies across treatment modalities (Nd: YAG membranotomy, pneumatic displacement, pars plana vitrectomy) and illustrates an important clinical gap as well as the need for development of the evidence-based management guidelines and treatment algorithms in sub-ILM hemorrhages.

## 7. Conclusions

Sub-internal limiting membrane hemorrhage represents a clinical entity characterized by a distinct, confined environment in which blood degeneration causes oxidative stress in proximity to the inner retina. This compartmentalization has direct implications for retinal damage, function, and visual prognosis. This review highlights gaps in research concerning molecular pathophysiology, imaging biomarkers, and clinical decision pathways for sub-ILM hemorrhage. Advances in molecular, biochemical, and cellular research have substantially improved understanding of mechanisms of retinal damage and further complications, such as, e.g., fibrosis, yet there is still a gap in fully understanding underlying processes, requiring further research. Advances in imaging techniques, such as optical coherence tomography, have improved diagnostic precision, but the absence of clear imaging criteria still limits proper classification and qualification to the proper modality of treatment. The present systematic review demonstrates that active interventions, e.g., Nd: YAG membranotomy, pneumatic displacement, and pars plana vitrectomy, accelerate hemorrhage clearance, limiting retinal exposure to toxic agents, which results in better retinal function and visual outcome in comparison to conservative treatment. However, there is a need to create standardized criteria for qualification to different modalities. The available evidence further suggests that delayed intervention is linked to a higher risk of retinal toxicity and compression injury, worse visual outcomes in patients, and a higher risk of complications such as proliferative vitreoretinopathy. These observations reinforce the rationale for earlier intervention, especially when the hemorrhage is foveal, large, or persistent. To conclude, developing evidence-based, consensus-driven guidelines is essential to optimize visual outcomes and prevent or diminish retinal injury.

## Figures and Tables

**Table 1:** Etiologies of sub-ILM hemorrhage: mechanisms, literature-based estimated frequency *, clinical features, typical presentation. * As sub-ILM hemorrhage is rare, and most published reports are case series, rather than epidemiologic studies, no formal population-based frequencies exist in the literature, according to the authors’ best knowledge.

Etiology	Estimated Frequency (% of Reported Sub-ILM Cases)	Pathophysiological Mechanism	Typical Patient Profile	Clinical Features	Prognostic Considerations
Valsalva retinopathy	35–45%	Sudden venous pressure rise, leading to a rupture of a superficial retinal vessel beneath ILM.	Usually overall healthy young adults with a history of heavy lifting/coughing/vomiting.	Sudden painless central scotoma; usually unilateral.	Excellent prognosis with decompression; Nd: YAG or PPV effective if dense.
Retinal arterial macroaneurysm (RAM) rupture	20–30%	Rupture of arterial macroaneurysm causing hemorrhage including sub-ILM.	Older hypertensive patients.	Acute unilateral vision loss; may show multilayer hemorrhage.	ILM-sparing PPV may preserve fovea; prognosis depends on hemorrhage layers.
Terson syndrome	10–15%	Acute intracranial pressure surge, causing cranial venous congestion, resulting in retinal vessel rupture under ILM.	Patients with subarachnoid hemorrhage (SAH) or severe head trauma; occurs often bilaterally.	Severe vision loss; often with associated vitreous hemorrhage.	More persistent hemorrhage; PPV commonly indicated.
Ocular trauma	10–20%	Blunt trauma, leading to shearing of the ILM and vessel rupture.	Often younger individuals; sports or accidents.	Acute visual acuity (VA) drop with coexisting traumatic signs.	Elevated risk of macular hole formation; PPV often required.
Blood dyscrasias (leukemia, anemia, thrombocytopenia)	3–8%	Vessel fragility or coagulopathy.	Patients with systemic hematologic disease.	Variable presentation; may be bilateral.	Management influenced by the degree of systemic stability.
Fragile neovascularization, e.g., proliferative diabetic retinopathy (PDR)	5–10%	Rupture of neovascular fronds.	Diabetic or ischemic retinopathy patients.	Acute/subacute VA loss; bleeding often multilayered.	Requires control of underlying neovascular disease; PPV if dense.
Idiopathic cases	<3%	Spontaneous microvascular rupture without clear underlying cause.	Any age; no systemic association.	Mild to moderate VA decline.	Often small and self-limiting.

**Table 2:** Chronology of the sub-internal limiting membrane hemorrhage: imaging biomarkers, biochemical correlates, intervention indications.

Hemorrhage Stage	Time Window	OCT Biomarkers	Biochemical State	Clinical Implications
Fresh hemorrhage	0–3 days	Smooth dome-shaped ILM elevation; homogeneous hyperreflective blood; intact ILM line.	Intact erythrocytes; oxyhemoglobin predominates; minimal iron release or reactive oxygen species (ROS) creation.	Best timing for Nd: YAG membranotomy with rapid visual recovery expected.
Early lysis phase	3–14 days	Increasing signal granularity; early internal heterogeneity or layering.	Hemoglobin oxidation to methemoglobin; onset of iron release; early ROS generation.	Toxicity begins to rise; pneumatic displacement ± tissue plasminogen activator (tPA) administration still feasible.
Oxidative/toxic phase	2–6+ weeks	Markedly heterogeneous or clotted appearance; persistent ILM elevation.	High Fe^2+^ load; Fenton reaction with ROS; marked Müller and retinal ganglion cell (RGC) oxidative stress.	Significant risk of inner retinal injury; PPV with ILM peeling increasingly favored.
Fibrotic/late stage	>6 weeks	Irregular ILM contour; tractional changes; organized clot.	Macrophage, fibroblast, and RPE-like cell proliferation; chronic degradation products present.	Lower visual prognosis; PPV often required; risk of PVR-like sequelae formation.

**Table 3:** Comparative summary of sub-ILM hemorrhage treatment modalities.

Treatment Modality	Mechanism of Action	Ideal Indications	Contraindications/Limitations	Typical Clearance Time	Expected Visual Outcome	Advantages	Risks
Observation	Slow spontaneous erythrocyte lysis and absorption without any iatrogenic intervention.	Small, extrafoveal hemorrhages with minimal visual impairment in medically fragile patients (e.g., leukemia).	Very slow clearance; prolonged exposure to iron/ROS-related retinal damage; increased risk of PVR-like changes.	Weeks–months.	Lowest mean VA improvement.	Non-invasive; no procedural risks.	Higher retinal toxicity risk; poor outcomes in dense or foveal hemorrhage.
Nd: YAG membranotomy	Creates focal ILM perforation which decompresses the sub-ILM compartment; blood drains into the vitreous.	Fresh (<1–2 weeks), non-clotted foveal hemorrhage with good posterior vitreous visualization.	Clotted or organized blood; very thick ILM; risk of off-target retinal impact.	Hours–days.	Rapid VA improvement.	Minimally invasive; immediate decompression.	Potential ILM/retinal injury; incomplete drainage; persistent premacular cavity possible.
Pneumatic displacement (±tPA)	Intravitreal gas shifts blood away from fovea; tPA dissolves clot when present.	Recent hemorrhage; moderately dense collections in patients with ability to hold posture.	Thick, chronic, highly organized clot (if no tPA); limited by need for patient positioning.	Days to 1–2 weeks.	Good VA recovery when performed early.	Minimally invasive; avoids vitrectomy; tPA effective for semi-clotted blood.	Gas-related complications; inconsistent efficacy; patient-dependent posturing adherence affecting outcome.
PPV without ILM peeling	Vitrectomy with mechanical evacuation of sub-ILM blood via incision/aspiration while preserving ILM.	Cases where ILM preservation is desirable, e.g., thin fovea, high macular hole (MH); some retinal arterial macroaneurysm (RAM) ruptures.	Residual blood/toxic products may remain; less complete decompression than ILM peel.	Immediate.	Good VA improvement; inferior to peeling in chronic or dense cases.	Protects native ILM; reduces risk of peeling-related trauma.	Persistent dome, incomplete clearance, possible need for secondary surgery.
PPV with buttonhole (fovea-sparing) ILM peeling	Selective ILM opening with “buttonhole” sparing of the foveal ILM while removing adjacent ILM to release hemorrhage.	RAM rupture; cases where preserving foveal ILM architecture is critical.	Technically demanding; not ideal for chronic or multilayer hemorrhage.	Immediate.	Reported to yield better foveal structural and visual outcomes than full peeling.	Decompresses hemorrhage while sparing fovea; reduces risk of iatrogenic MH.	Requires surgical expertise; may not fully clear toxic compartment if bleeding is chronic.
PPV with conventional ILM peeling	Complete removal of ILM and evacuation of the blood; completely eliminates confined compartment and toxic breakdown products.	Dense or long-standing, clotted macular hemorrhage; chronic or recuring cases; multilayer hemorrhages; Terson syndrome.	Surgical risks; not preferred when foveal ILM preservation is needed.	Immediate.	Highest and most reliable VA gains; most complete anatomic result.	Definitive clearance of iron/ROS burden; prevents PVR-like sequelae; effective regardless of chronicity.	Risk of retinal trauma, macular hole, retinal detachment (RD), cataract; most invasive option.

## Data Availability

No new data were created or analyzed in this study. Data sharing is not applicable to this article.

## Abbreviations

The following abbreviations are used in this manuscript:

AF	Autofluorescence
cGMP	Cyclic Guanosine Monophosphate
EAAT	Excitatory Amino Acid Transporter
ECM	Extracellular matrix
ILM	Internal limiting membrane
Nd: YAG	Neodymium: yttrium–aluminum–garnet laser
NIR	Near-infrared reflectance
NFL	Nerve fiber layer
SD-OCT	Spectral domain optical coherence tomography
PPV	Pars plana vitrectomy
PVD	Posterior vitreous detachment
PVR	Proliferative vitreoretinopathy
RAM	Retinal arterial macroaneurysm
RD	Retinal detachment
RGC	Retinal ganglion cell
RNFL	Retinal nerve fiber layer
ROS	Reactive oxygen species
SAH	Subarachnoid hemorrhage
tPA	Tissue Plasminogen Activator
VA	Visual acuity
VH	Vitreous hemorrhage
